# Nephrotoxicity in survivors of Wilms' tumours in the North of England

**DOI:** 10.1038/sj.bjc.6600608

**Published:** 2002-11-04

**Authors:** S Bailey, A Roberts, C Brock, L Price, A W Craft, R Kilkarni, R E J Lee, A W Skillen, R Skinner

**Affiliations:** Department of Child Health, University of Newcastle upon Tyne, Newcastle upon Tyne, UK; Department of Clinical Biochemistry, University of Newcastle upon Tyne, Newcastle upon Tyne, UK; Department of Radiology, Royal Victoria Infirmary, Newcastle upon Tyne, UK; Northern Centre for Cancer Treatment, Newcastle upon Tyne, UK

**Keywords:** Wilms' tumour, nephrotoxicity, glomerular, proximal tubule, distal tubule

## Abstract

One aspect of concern for survivors of Wilms' tumour has been the late outcome in terms of renal function. Previous studies have documented low glomerular filtration rate and high blood pressure in some patients. Furthermore, disorders in tubular function (especially urinary concentration defects) have been suggested but not confirmed in small studies. The aim of this study was to determine the prevalence and nature of subclinical and overt glomerular, proximal and distal renal tubular toxicity in a population based cohort of survivors of Wilms' tumour. Forty patients (24 female) with a median age of 4.3 years (3 months–11.8 years) at diagnosis were studied. Median follow-up was 8.8 (range 0.06–27.5) years. Glomerular filtration rate was measured by ^51^Cr-EDTA plasma clearance, proximal tubular function by electrolyte fractional excretions, urine excretion of low molecular weight proteins (retinol-binding protein) and renal tubular enzymes (alanine aminopeptidase; N-acetylglucosaminidase) and distal tubular function by the osmolality of the first two urines of the day on 3 consecutive days. Renal size (ultrasound) and blood pressure were also measured. Mean (range) glomerular filtration rate was 100 (61–150) ml min^−1^ 1.73 m^−2^. Nine were below the reference range for healthy individuals with two kidneys. Most serum electrolyte concentrations (sodium, potassium, chloride, calcium, magnesium and phosphate) fell within the normal range for age, as did the fractional excretions. The values that fell outside the normal range were only marginally abnormal. Subclinical measures of tubular toxicity (retinal-binding protein, alanine aminopeptidase, N-acetylglucosaminidase) were abnormal in only four patients. Thirty-seven patients achieved maximal urine osmolalities ⩾800 mOsm kg^−1^, but three failed to achieve this value even after DDAVP administration. Two patients had evidence of increased urinary albumin excretion. Compensatory renal hypertrophy was seen in all but two patients, but blood pressure was within normal limits in all patients. Current and past treatment for Wilms' tumour does not have any clinically important nephrotoxic effect in the majority of patients. This finding will enable paediatric oncologists to reassure patients and parents that treatment for Wilms' tumour rarely causes long-term renal impairment.

*British Journal of Cancer* (2002) **87**, 1092–1098. doi:10.1038/sj.bjc.6600608 www.bjcancer.com

© 2002 Cancer Research UK

## 

Nephroblastoma or Wilms' tumour is one of the commonest solid malignancies in childhood, with an incidence of one in 10 000 live births (Mitchell, 1997). This triphasic tumour occurs usually between the ages of 2 and 7 years (Breslow and Beckwith, 1982) with an equal sex incidence. Nephrectomy was first described as treatment for this tumour in the 1940s (Priestly and Shulte, 1942) and since that time the adjuvant treatment has undergone a number of changes. Radiotherapy and chemotherapy have been used, often in combination. Chemotherapy has been used both pre- and post-nephrectomy. Advances in therapy have resulted in a greatly improved survival rate for all stages of the disease. In 1910 virtually all children with Wilms' tumour died; by 1950 with the advent of safe anaesthesia and radiotherapy survival had risen to 50%, and current treatment leads to survival in excess of 90% (Mitchell, 1997).

With this excellent prognosis there has been a shift towards attempts to reduce the late toxicity of treatment of these children without compromising their prognosis. One such area of concern has been functional impairment of the remaining kidney. Reduction of the duration or intensity of chemotherapy and radiotherapy occurred in successive studies over the last 30 years. Nephron sparing surgery rather than total nephrectomy to reduce the impact of possible late effects on a solitary kidney has been suggested.

There is still relatively little data on the function of the remaining kidney after treatment for Wilms' tumour. Reported effects after treatment for Wilms' tumour have included reduction in glomerular filtration rate (GFR) (Walker *et al*, 1982; Robitalle *et al*, 1985; Levitt *et al*, 1992), microalbuminuria (Levitt *et al*, 1992; di Tullio *et al*, 1996) (rarely resulting in hyperfiltration induced glomerulosclerosis and subsequent renal failure), failure of compensatory hypertrophy of the remaining kidney (Wikstad *et al*, 1986; Makipernaa *et al*, 1991; Levitt *et al*, 1992) and hypertension (Levitt *et al*, 1992).

The aim of this study was to document the prevalence and nature of renal toxicity in survivors of children treated for Wilms' tumour.

## PATIENTS AND METHODS

### Patients

Fifty-eight survivors of children who presented with Wilms' tumour between 1966 and 1998 in the North of England were identified from the North of England Young Persons Malignant Disease Registry. Forty patients were studied (69%); of the remaining 18 patients 13 had moved from the area, three declined the invitation to participate, and their general practitioners suggested that it was inappropriate to approach two patients.

Out of the forty patients studied 24 were female (60%). The median age at diagnosis was 4.3 (years) (3 months–11.8 years). The median duration of follow up was 8.8 years (range 0.06–27.5). The right kidney was affected in 24 patients (61.5%). Eighteen patients (45%) had stage I disease, nine (22.5%) stage II disease, seven (17.5%) stage III disease, five (12.5%) stage IV disease and one (2.5 %) stage V disease. Out of the 18 patients not studied seven (39%) had stage I disease, four (22%) stage II disease, six (33%) stage III disease and one (5.5%) had stage IV disease. The median age at diagnosis of these patients was 3.0 years (range 0.05–7.2). Two of these patients were diagnosed during the 1960's, nine during the 1970's, four during the 1980's and two during the 1990's. Out of the eight patients not studied and treated before the mid 1970's five had whole abdominal radiotherapy.

### Treatment

Children were treated with a combination of surgery (all patients), radiotherapy (19 patients – 47.5%) and chemotherapy (all patients) (Table 1

**Table 1:**
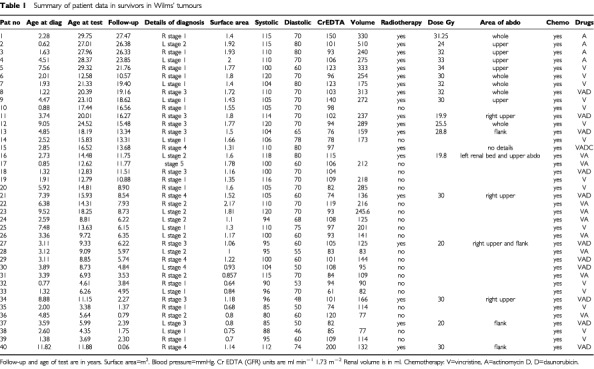
Summary of patient data in survivors in Wilms' tumours

). Surgery involved unilateral nephrectomy in all but one patient with stage 5 disease who had a wedge resection of one kidney and a complete removal of the other. Radiotherapy varied during the time period covered by the study. In the earlier years (prior to 1974) randomisation between whole abdominal radiotherapy and flank radiotherapy was performed but after this date flank radiotherapy was used in most patients. Out of the 19 patients receiving radiotherapy, five had whole abdominal radiotherapy, and 14 had flank radiotherapy (median dose of 3.3 Gy to remaining kidney). The median dose to the kidney bed (i.e. on tumour side) was 30 (range 19.8–34) Gy given in a median of 16 fractions (range 10–22). Three patients also had radiotherapy to their lungs as well as their abdomens. Chemotherapy was given to all patients, four had actinomycin alone (10%), 16 had vincristine alone (40%), eight had vincristine and actinomycin (20%), 11 had vincristine, actinomycin and doxorubicin (27.5%) and one patient had vincristine, actinomycin, doxorubicin and cyclophosphamide (2.5%). Most patients were treated according to national protocols, i.e. MRC 1 from 1970–1974, MRC 2 from 1974–1980, UKCCSG Wilms 1 from 1980–1986, UKCCSG Wilms 2 from 1986–1991 and UKCCSG Wilms 3 from 1991 onwards.

### Renal investigations

Glomerular filtration rate was measured by ^51^Cr-EDTA plasma clearance.

Proximal tubular function was evaluated by plasma electrolyte concentrations and fractional excretions (sodium, potassium, chloride, magnesium, phosphate, calcium and glucose). Fractional excretion was calculated by the formula: (urine electrolyte concentration/serum electrolyte concentration)×(serum creatinine concentration/ urine creatinine concentration)×100. An increased fractional excretion is only significant if the corresponding serum electrolyte concentration is reduced. The Tm_p_/GFR is a measure of the renal tubular threshold for phosphate and is defined as plasma phosphate–(urine phosphate×plasma creatinine)/urine creatinine.

Sub-clinical measures of renal tubular impairment were measured by urine concentrations of a low molecular weight protein (retinol-binding protein, RBP) and of two renal tubular enzymes (alanine aminopeptidase, AAP and N-acetylglucosaminidase, NAG).

Distal tubular function was evaluated by the osmolality of the first two urines of the day on 3 consecutive days. If a maximal urine concentration of 800 mOsm kg^−1^ was not achieved then DDAVP was administered and the subsequent maximal urine concentration recorded. Fasting serum arginine-vasopressin levels were also measured.

Urinary albumin excretion was determined using an untimed urine sample and applying the formula urine albumin (mg)/urine creatinine (mmol).

Renal size (measured by ultrasound) was evaluated using the formula: length×width×(longitudinal+transverse depth)/2×0.523 (Wikstad *et al*, 1986).

Blood pressure (BP) was measured using a mercury sphygmomanometer. The current BP of patients not studied was obtained in 14 out of 18 patients by contacting their general practitioners.

Ethical Approval was obtained from the Newcastle and North Tyneside Health Authority (Min. ref. 94/282).

## RESULTS

### Glomerular

**Figure 1:**
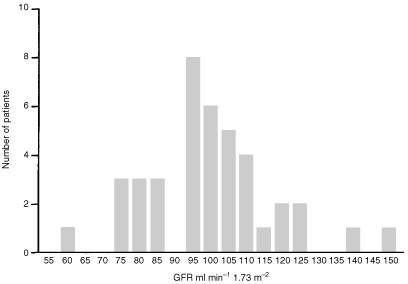
Frequency histogram of glomerular filtration rate. The median value was 100 ml min^−1^ per 1.73 m^−2^ with a range of 61–150 ml min^−1^ per 1.73 m^−2^.

**Figure 2:**
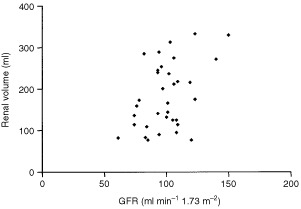
Renal volume (ml) is compared with GFR (ml min^−1^). There is a correlation between GFR and renal volume in the remaining kidney (*r*=0.37, *P*=0.03).

Glomerular filtration rate was measured in all 40 patients with a median of 100 ml min^−1^ 1.73 m^−2^ (range 61–150). Nine patients had a GFR below the reference range for healthy individuals with two kidneys (>90 ml min^−1^ 1.73 m^−2^), and four had a GFR below 80 ml min^−1^ 1.73 m^−2^ (Figure 1

and Table 1). There appeared to be a weak but significant correlation between renal volume and GFR in the study patients (*r*=0.37, *P*=0.03) (Figure 2

). Those patients with larger renal volumes tended to have higher GFR. This may suggest a degree of hyperfiltration of the remaining kidney. There was no significant difference in the GFR of those patients who had received radiotherapy compared to those that did not (*P*=0.2, Mann–Whitney *U*-test).

### Proximal tubular function

Serum electrolyte concentrations are shown in Table 2

**Table 2:**
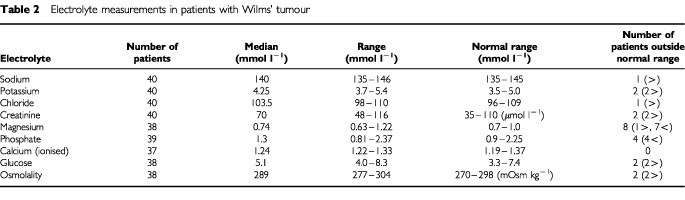
Electrolyte measurements in patients with Wilms' tumour

and their fractional excretions in Table 3

**Table 3:**
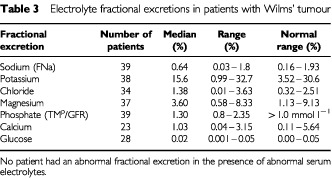
Electrolyte fractional excretions in patients with Wilms' tumour

. All those values that fell outside the normal range were only marginally abnormal. Those with fractional excretions outside the reference ranges all had normal serum electrolyte concentrations. Out of the eight serum magnesium concentrations that fell outside the reference range, seven were only marginally low but the other was moderately elevated; in the case of four abnormal serum phosphate concentrations, three were marginally lower and one higher than the reference range. Out of the seven patients with marginally decreased serum magnesiums, four also had marginally decreased Tm_p_/GFR's and two of the eight also had marginally decreased serum phosphates. Five of the seven patients with decreased magnesium concentrations received radiotherapy during their treatment. None of these patients had abnormal urinary enzyme excretion.

### Sub-clinical proximal tubular toxicity

**Figure 3:**
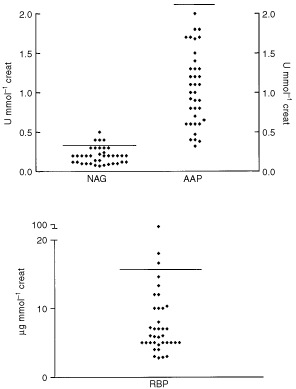
Urinary excretion of RBP, NAG and AAP. The median urine RBP concentration was 5.95 μg mmol^−1^ Cr (range 0.45–63). The median urine AAP concentration was 1 U mmol^−1^ Cr (range 0.32–2). The median urine NAG concentration was 0.2 U mmol^−1^ Cr (range 0.07–0.5). The solid horizontal lines represent the upper limit of the reference range.

The median urine RBP concentration was 5.95 μg mmol^−1^ Cr (range 0.45–63), three values were outside the normal range. The median urine AAP concentration was 1 U mmol^−1^ Cr (range 0.32–2) with no values being outside the normal range. The median urine NAG concentration was 0.2 U mmol^−1^ Cr (range 0.07–0.5), four values were outside the normal range (Figure 3

).

### Distal tubular function

Maximal urine concentrations of more than 800 mOsm kg^−1^ were achieved in 37 patients with a median value of 859 mOsm kg^−1^ and a range of 182–1251 mOsm kg^−1^. In three patients the maximal concentration did not reach 800 mOsm kg^−1^ despite DDAVP administration. These three patients had maximal concentrating ability of 520, 640 and 560 mOsm kg^−1^ despite the administration of DDAVP. The GFR in these patients were 109, 109, 100 ml min^−1^ 1.73 m^−2^ respectively.

Serum vasopressin concentrations were measured in 31 patients and ranged between 0.4 and 9.8 pg ml^−1^ (median=1.8 pg ml^−1^), four fell marginally outside the normal reference range (three lower and one higher) corrected for plasma osmolality.

### Urine albumin excretion

Urine albumin/creatinine ratio was measured in 36 patients and ranged from 0.02–46.7 mg mmol^−1^ (median 1.43) (normal range=<10 mg mmol^−1^). Increased albumin excretion was found in two patients (5%). These patients had no other abnormality in renal function.

### Blood pressure

**Figure 4:**
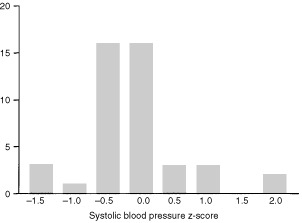
Frequency histogram of systolic blood pressure. The z-scores related to the medians for age ranged between −1.51 and 1.8 (median −0.24).

Systolic BP ranged between 80 and 120 mmHg (median 108 mmHg). The z-scores related to the medians for age (Task Force, 1987) ranged between –1.51 and 1.80 (median –0.24) (Figure 4

). Diastolic BP ranged between 46 and 80 mmHg (median 70 mmHg). The z-scores ranged between –1.57 and 1.27 for age (median 0.23). There were no abnormal systolic or diastolic blood pressures in the patients studied (Table 1). The systolic BP for 14 patients unable to take part in the study ranged between 100 and 140 mmHg (median 120 mmHg) and the diastolic between 60 and 90 mmHg (median 76 mmHg). One of these 14 individuals was being treated for hypertension and four out of 14 had systolic BPs over 130 mmHg but less than 140 mmHg. These tended to be patients who were treated in the early part of the study period. We were unable to obtain information about the BP of four patients.

### Renal size

Renal size of 38 patients was recorded using the method described by Dinkel (1985). Renal volume ranged from 77.3–510 ml (median=173 ml) (Table 1). Compensatory hypertrophy (kidney length greater than two standard deviations above the mean for age and weight) was seen in 36 patients, the other two patients had renal sizes above the median for their weight.

### Influence of age

Four patients were less than 1 year old and 10 were less than 2 years old at diagnosis; their results did not differ significantly from the rest of the patients studied.

### Influence of treatment

Abnormalities in renal function were too infrequent to evaluate the relevance of different components of treatment in the development of renal impairment (Table 1).

## DISCUSSION

With the survival of children for Wilms' tumour being about 90% for all stages combined the emphasis of treatment for this condition has shifted to minimise the toxicity of treatment while not compromising efficacy. Damage to the surviving kidney is a potential concern among those treating children with Wilms' tumour and it is important to fully document this effect and determine its significance in clinical practice.

There have been a number of studies with varying results looking at BP in survivors of Wilms' tumours (Table 4

**Table 4:**
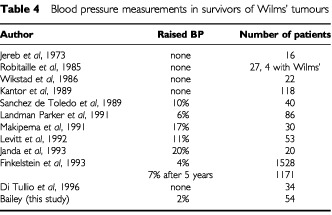
Blood pressure measurements in survivors of Wilms' tumours

), the largest of these being the NWTS study in which 7% of patients had an increased systolic BP after 5 years and the same number had an increase in diastolic BP (Kantor *et al*, 1989). Several studies showed no increase in blood pressure and others varied from 1–25%. However, the numbers of most studies are small.

Glomerular filtration rate has been measured in various series after treatment for Wilms' tumour, all of which show a small number of children with a low GFR (Table 5

**Table 5:**
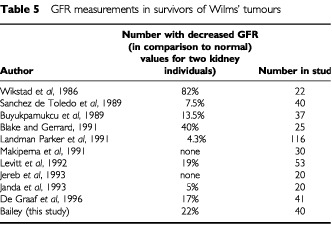
GFR measurements in survivors of Wilms' tumours

). The normal values that have been used in most cases are those for children with two kidneys. The median GFR is marginally higher than that described in other studies for nephrectomised patients with both malignant and non malignant conditions (90 ml min^−1^ 1.73m^−2^) (Walker *et al*, 1982; Robitalle *et al*, 1985). The reduction in GFR after treatment does not appear to be a major clinical problem. It has been suggested (de Graaf *et al*, 1996) the GFR is reduced in those children who have received radiotherapy compared to those who had chemotherapy alone. However this was not the case in this study. A reason for this may be the longer follow up period in this study (median 158 months *vs* 13 months). The median GFR in this study was higher than expected with a number of patients with values in excess of 110 ml min^−1^ 1.73m^−2^. Although renal plasma flow with and without protein loading were not performed the suggestion of hyperfiltration may be made on the basis of GFR alone (Donckerwolke and Coppes, 2001). Although no significant renal damage was noted in these patients at the present time this may in fact develop in the future with a longer period of follow-up.

A number of studies have been reported with regard to various aspects of proximal tubular function and proteinuria in survivors of Wilms' tumour (Table 6

**Table 6:**
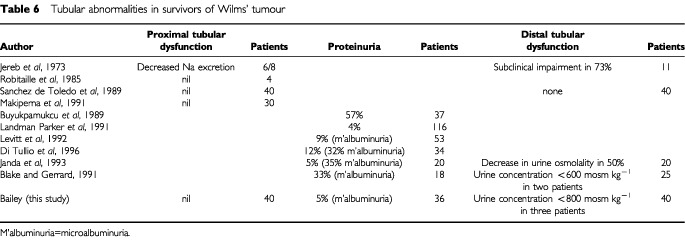
Tubular abnormalities in survivors of Wilms' tumour

). The vast majority of studies have not comprehensively studied the range of possible complications relating to the proximal tubule. While the results vary, there appears to be some degree of proteinuria (usually microalbuminuria) in a significant minority of patients, although this does not seem to cause clinical problems. Although there was some overlap of electrolyte abnormalities in individual patients none of these patients had more than two minor abnormalities. Proximal tubular abnormalities would be unexpected in children having had treatment for Wilms' tumour as the chemotherapy does not involve drugs which are known to have a major effect on proximal tubular function.

Distal tubular function has rarely been studied (Table 6). While some abnormalities were detected these appear to be of little clinical relevance to the patients. Impaired compensatory hypertrophy in the remaining kidney has been found in half the patients in one study but not in two others (Table 7

**Table 7:**
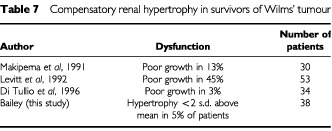
Compensatory renal hypertrophy in survivors of Wilms' tumour

).

The limited number of previous studies has indicated that hypertension occurs in 7% (Finkelstein *et al*, 1993) of children treated for Wilms' tumour although in a study of 119 survivors Kantor found no hypertensive patients (Kantor *et al*, 1989). The results of our study are consistent with this finding that hypertension in survivors of Wilms' tumour is not a common clinical problem. Compensatory renal hypertrophy was found in all but two of our patients. These two patients did not have any other signs of renal dysfunction. They were not under 2 years old at diagnosis (Levitt *et al*, 1992) and their treatment was not different from other patients. The results of our study that lack of compensatory hypertrophy is not a significant problem in survivors of Wilms' tumour is in contrast to suggestions by others (Green and Jaffe, 1979; Zuchelli *et al*, 1983). The type of treatment, including whether or not radiotherapy was given, did not appear to influence renal dysfunction; this is in keeping with previous studies (Jereb *et al*, 1973; Luttenegger *et al*, 1975; Walker *et al*, 1982). The type of radiotherapy (whole abdomen *vs* flank) did not appear to exert an influence on toxicity.

Although there was some evidence of minor renal dysfunction in some of our patients, these abnormalities appeared to be isolated. Three patients had evidence of minor impairment of renal concentrating ability but not of a degree sufficient to cause clinical problems. Nine patients had a marginally reduced GFR but again the magnitude of these abnormalities was small and unlikely to cause clinical sequelae. There was no evidence of clinically relevant proximal renal dysfunction in any patient. Proximal tubular function of survivors of Wilms' tumours has not been evaluated in this degree of detail before, so direct comparison is not possible.

Hyperfiltration and subsequent development of focal glomerulosclerosis and renal failure is a concern of many paediatric oncologists treating Wilms' tumour. Increased excretion of albumin has been described in up to a third of patients surviving Wilms' tumour (Levitt *et al*, 1992; di Tullio *et al*, 1996) but was rare in our patients. Although the two patients with microalbuminuria may develop late glomerular problems, none are yet apparent 5 and 6 years after treatment. The significance of the increase in GFR in some of our patients is unknown but may lead to hyperfiltration injury and resultant renal damage in the future.

Although the 18 patients not studied were mainly from the early part of the study period, this may have led to some bias of the results. The age at diagnosis and the stage however were similar to that of the population studied.

In conclusion, we have shown in a population-based study that there is no clinically significant toxicity associated with either past or present treatment for this condition. This should help paediatric oncologists to reassure patients and parents that treatment for Wilms' tumour is unlikely to cause long-term renal impairment and reassure paediatric surgeons that nephron sparing surgery is not necessarily important in these children.
